# Supplementary material for: Serial cryoFIB/SEM Reveals Cytoarchitectural Disruptions in Leigh Syndrome Patient Cells
Source: Structure. 2021 Jan 7;29(1):82–87.e3. doi: 10.1016/j.str.2020.10.003 (PMC7802768; doi:10.1016/j.str.2020.10.003)
Supplement: Document S1. Figures S1–S3 and Table S1 [file mmc1.pdf]

Structure, Volume *29*

Supplemental Information

Serial cryoFIB/SEM Reveals Cytoarchitectural Disruptions in Leigh Syndrome Patient Cells

Yanan Zhu, Dapeng Sun, Andreas Schertel, Jiying Ning, Xiaofeng Fu, Pam Pam Gwo, Alan M. Watson, Laura C. Zanetti-Domingues, Marisa L. Martin-Fernandez, Zachary Freyberg, and Peijun Zhang

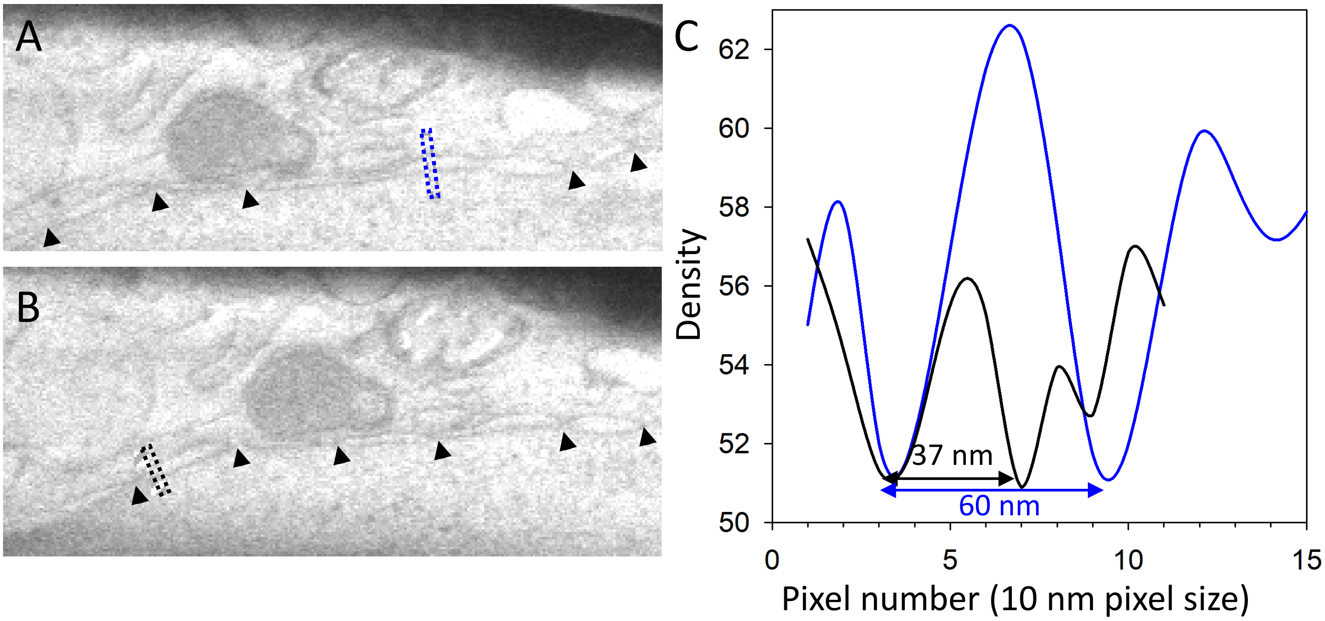

Figure S1 | Resolution assessment. A-B) Representative cryoSEM images from a stack of 2018 serial micrographs recorded from a patient fibroblast cell. Black arrow heads indicate nuclear pores. Blue and black boxes enclose areas for density profile. C) Density profile plots of the nuclear membranes. The blue line from the area shown in (A) and the black line from the area shown in (B). The separation between the two membranes is 37 nm in (B). Related to Figure 2.

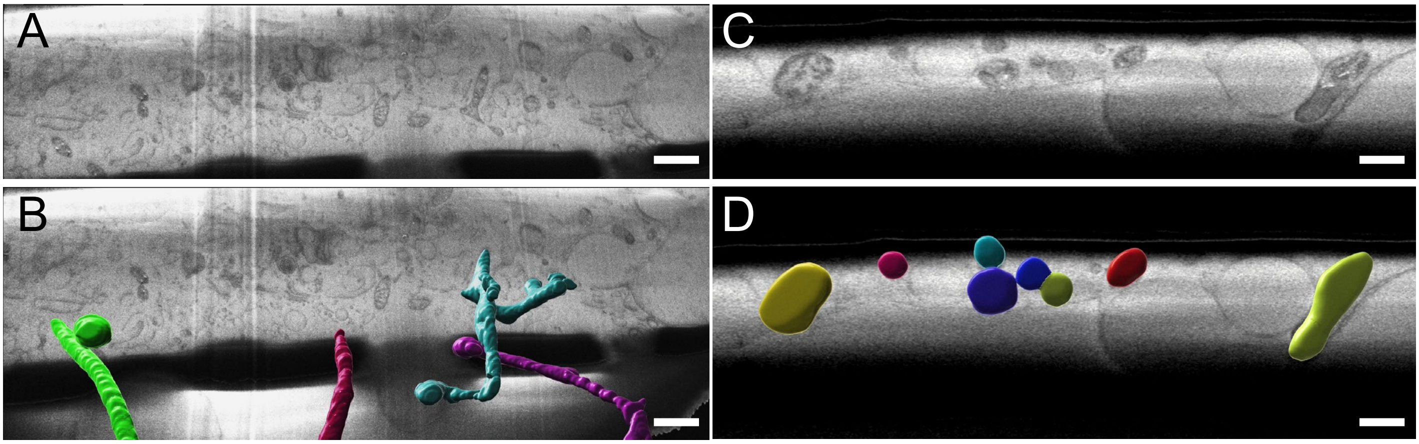

Figure S2 | Close association of mitochondria in control (A-B) and patient (C-D) cells. Mitochondria were manually segmented from control (A) and patient (C) datasets using NIS Elements (Nikon). The segmented images were overlaid with the RAW data in Imaris 9.5 (Bitplane) where the segmented areas were transformed into objects using surfacing tools (B and D, also see Movie S7 and S8). Independent objects are signified by a unique color. Scale bars, 1 µm in a & b, 0.3 µm in C & D. Related to Figure 3.

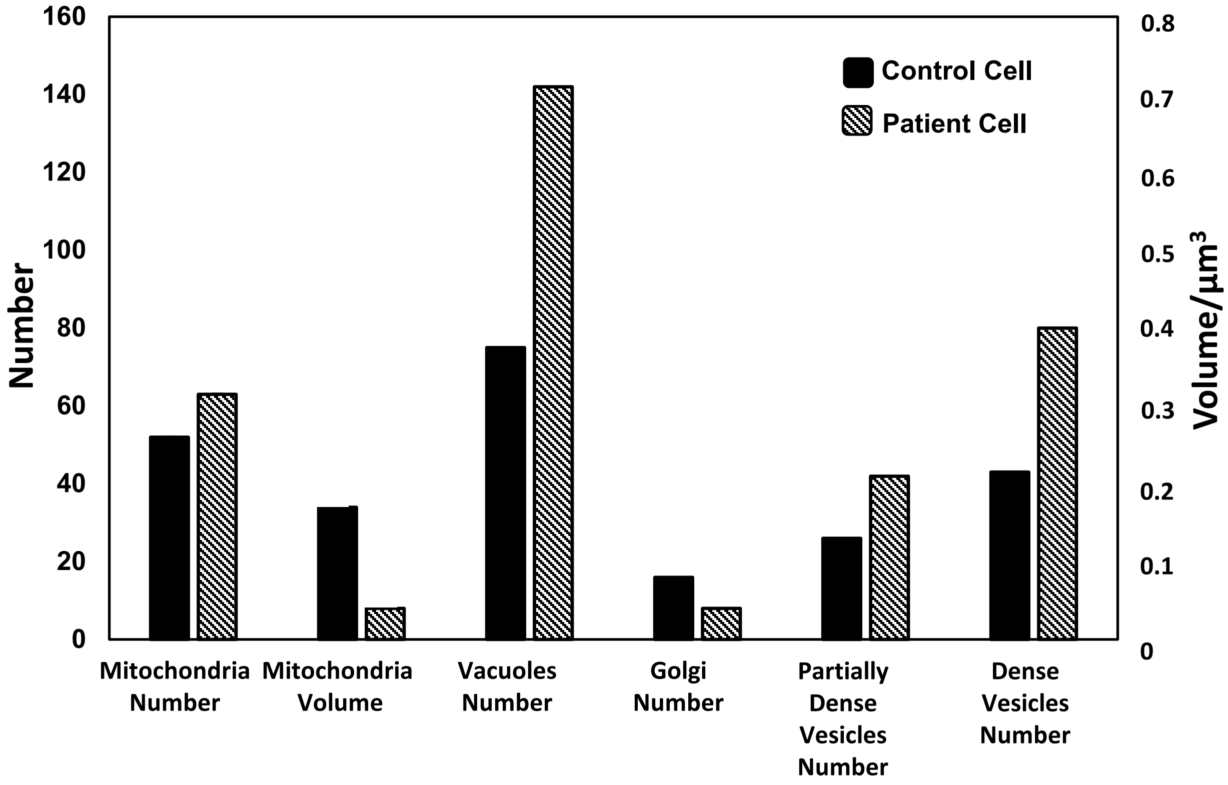

Figure S3 | Number and volume of different organelles in control and patient cells. Related to Figure 3.

Table S1: Summary of serial cryoFIB/SEM parameters. Related to STAR☆Methods, Methods Details, Serial cryoFIB/SEM section.

	FIB Probe current (pA)	SEM Probe current (pA)	SEM acceleration potential (keV)	Dose pA*nS /Å²	Dwell time (ns)	Line averagin g count	Pixel size x-y-z (nm)	Image size x-y (pixel)	No. Slices	Volume (μm³)	Time (h:m)
**Patient**	700	35	2.3	21.3	100	61	10.5 x 10.5 x 21.0	4096 × 3072	2018	50,784	17:20
**Control**	700	59	1.9	22.4	200	19	10.0 x 10.0 x 20.0	3072 × 1150	575	4,062	5:40
