# Supplementary material for: Serial cryoFIB/SEM Reveals Cytoarchitectural Disruptions in Leigh Syndrome Patient Cells
Source: Structure. 2021 Jan 7;29(1):82–87.e3. doi: 10.1016/j.str.2020.10.003 (PMC7802768; doi:10.1016/j.str.2020.10.003)
Supplement: Document S2. Article plus Supplemental Information [file mmc8.pdf]

Structure

Short Article

Serial cryoFIB/SEM Reveals Cytoarchitectural Disruptions in Leigh Syndrome Patient Cells

Graphical Abstract

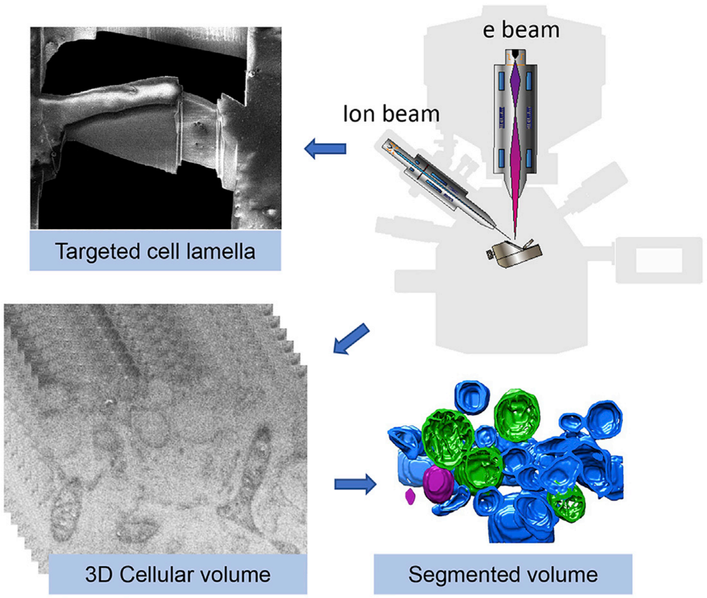

Authors

Yanan Zhu, Dapeng Sun, Andreas Schertel, ..., Marisa L. Martin-Fernandez, Zachary Freyberg, Peijun Zhang

Correspondence

peijun@strubi.ox.ac.uk

In Brief

Serial cryoFIB/SEM offers new opportunities for structural analysis of cells and tissues under near-native conditions. Zhu et al. developed and optimized a serial cryoFIB/SEM volume imaging workflow for visualization of entire vitreous cells in 3D, and demonstrated its potential for clinical and pathological applications with primary Leigh syndrome patient cells.

Highlights

- Developed and optimized a serial cryoFIB/SEM volume imaging workflow
- Visualized the 3D structure of an entire cell under native conditions
- Revealed a disruption of cellular structures in primary LS patient fibroblasts
- Demonstrated the potential for clinical phenotyping of pathogenic tissues

Zhu et al., 2021, Structure *29*, 82–87
January 7, 2021 © 2020 The Authors. Published by Elsevier Ltd.
https://doi.org/10.1016/j.str.2020.10.003

CellPress
OPEN ACCESS

Structure

Short Article

Serial cryoFIB/SEM Reveals Cytoarchitectural Disruptions in Leigh Syndrome Patient Cells

Yanan Zhu,[1,8] Dapeng Sun,[2,8] Andreas Schertel,[3] Jiying Ning,[2] Xiaofeng Fu,[2] Pam Pam Gwo,[4] Alan M. Watson,[5] Laura C. Zanetti-Domingues,[6] Marisa L. Martin-Fernandez,[6] Zachary Freyberg,[4,5] and Peijun Zhang[1,2,7,9,*]
[1]Division of Structural Biology, Wellcome Trust Centre for Human Genetics, University of Oxford, Oxford OX3 7BN, UK
[2]Department of Structural Biology, University of Pittsburgh School of Medicine, Pittsburgh, PA 15260, USA
[3]Carl Zeiss Microscopy GmbH, Zeiss Customer Center Europe, Carl-Zeiss-Strassee 22, 73447 Oberkochen, Germany
[4]Department of Psychiatry, University of Pittsburgh, Pittsburgh, PA 15213, USA
[5]Department of Cell Biology, University of Pittsburgh, Pittsburgh, PA 15213, USA
[6]Central Laser Facility, Research Complex at Harwell, STFC Rutherford Appleton Laboratory, Harwell Oxford, Didcot, Oxford OX11 0QX, UK
[7]Electron Bio-Imaging Centre, Diamond Light Source, Harwell Science and Innovation Campus, Didcot OX11 0DE, UK
[8]These authors contributed equally
[9]Lead Contact
*Correspondence: peijun@strubi.ox.ac.uk
https://doi.org/10.1016/j.str.2020.10.003

SUMMARY

The advancement of serial cryoFIB/SEM offers an opportunity to study large volumes of near-native, fully hydrated frozen cells and tissues at voxel sizes of 10 nm and below. We explored this capability for pathologic characterization of vitrified human patient cells by developing and optimizing a serial cryoFIB/SEM volume imaging workflow. We demonstrate profound disruption of subcellular architecture in primary fibroblasts from a Leigh syndrome patient harboring a disease-causing mutation in USMG5 protein responsible for impaired mitochondrial energy production.

INTRODUCTION

Cryoelectron tomography (cryoET), with subtomogram averaging, has emerged as a powerful method for visualizing heterogeneous structures and *in situ* specimens at subnanometer resolutions (Himes and Zhang, 2018; Sutton et al., 2020; Zhang, 2019). However, due to limited penetrance of the electron beam in thicker regions of cells (Lucic et al., 2013; Wang et al., 2012), its utility is limited to very thin samples (<300 nm), such as thin regions of the cell periphery or cell lamella by cryo-focused ion beam (cryoFIB) thinning. On the other hand, serial FIB/scanning electron microscopy (SEM) has been rapidly adopted as a technique for generating large 3D volumes of cells and tissue constituents, which have been fixed (cryo or chemically), dehydrated, resin-embedded, and stained for imaging contrast (Kizilyaprak et al., 2019; Schirra and Zhang, 2014; Steyer et al., 2019). Its application to vitreous biological samples, namely serial cryoFIB/SEM, involves many challenges associated with low-contrast (no staining) and low-dose (radiation sensitive) imaging. Examples of serial cryoFIB/SEM showed its potential for studying whole-mount plunge-frozen and high-pressure frozen cells and tissues (Akiva et al., 2019; Schertel et al., 2013; Sviben et al., 2016; Vidavsky et al., 2015, 2016; Wu et al., 2020). We now explore this new capability for pathologic characterization of Leigh syndrome (LS) patient cells harboring a disease-causing mutation in USMG5 protein responsible for impaired mitochondrial energy production.

The primary role of mitochondria is to generate energy in cells through mitochondrial oxidative phosphorylation (OXPHOS) (Lake et al., 2015). OXPHOS deficiency leads to mitochondrial diseases, including LS, a devastating neurological disorder and the most common mitochondrial disease in children (Sofou et al., 2014). LS is genetically heterogeneous with more than 90 nuclear or mitochondrial genes implicated in its pathogenesis (Chang et al., 2020; McCormick et al., 2018). Virtually all of these genes encode the mitochondrial respiratory complex machinery required for energy generation through OXPHOS (Barca et al., 2018), including those regulating the structure and assembly of complex V (ATP synthase). Classical transmission electron microscopy of thin tissue sections from LS patients is typically used to diagnose mitochondrial disease, revealing abnormality of the structure of mitochondria (Lee et al., 2016). Disease-causing mutations, such as (T8993G-1) in cytochrome *c* oxidase (complex IV) and in SURF1 (a complex IV protein) were shown to lead to ultrastructural changes in mitochondria and, in the case of SURF1, also aggregation of abnormal intracellular inclusions (Makino et al., 2000; Pronicki et al., 2008). Recently a genetic study identified a novel pathogenic mutation (c.87 + 1G > C), in the *USMG5* gene that results in autosomal recessive LS (Barca et al., 2018). The mutation abolishes the canonical GT splice site donor of exon 4 of *USMG5* and produces aberrant transcripts that are degraded via nonsense-mediated decay with >90% loss of USMG5 expression (Barca et al., 2018). USMG5, also known as DAPIT (diabetes-associated protein in

 © 2020 The Authors. Published by Elsevier Ltd.
This is an open access article under the CC BY license (http://creativecommons.org/licenses/by/4.0/).

CellPress
OPEN ACCESS

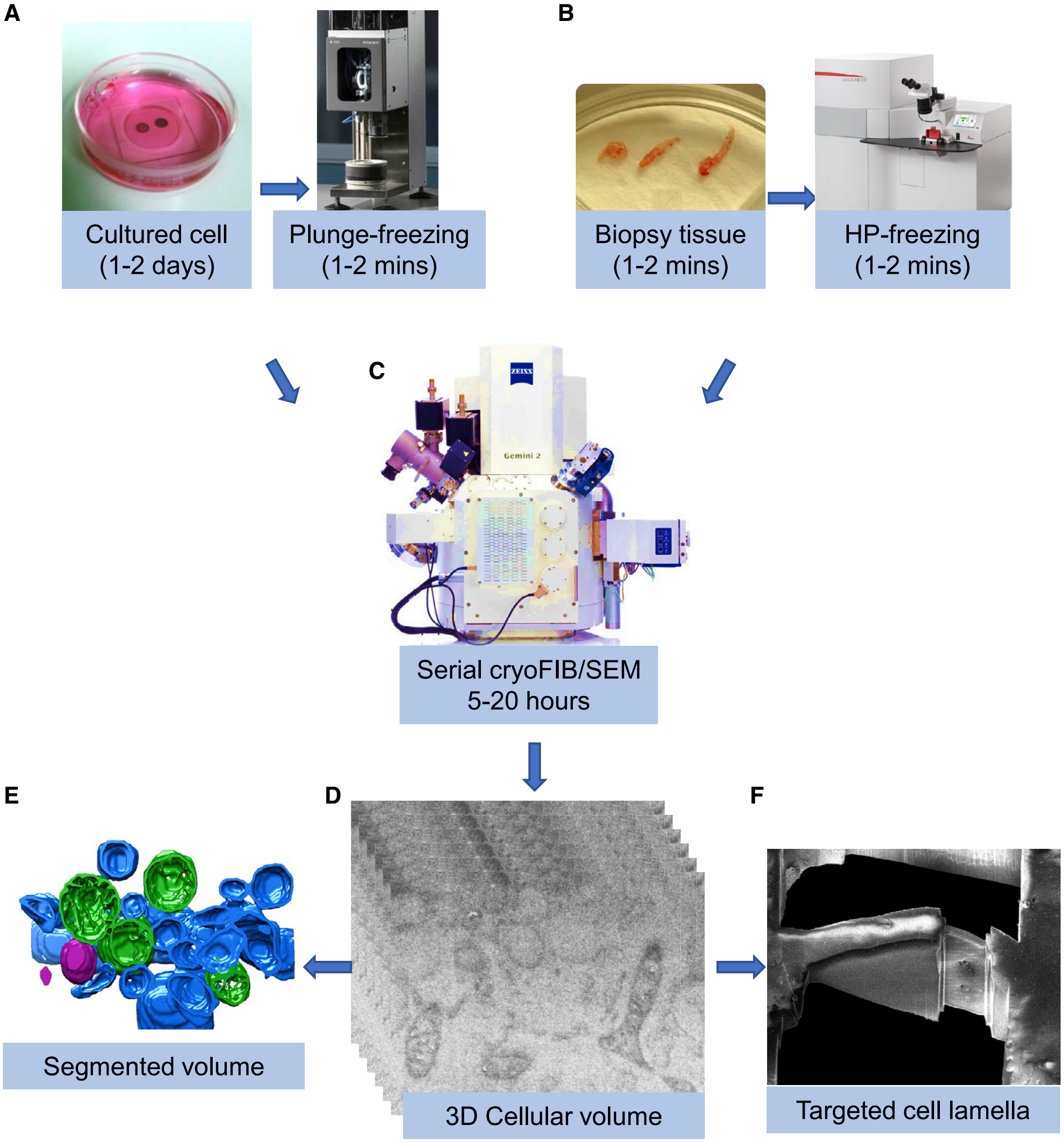

Figure 1. A Workflow for Serial cryoFIB/SEM Volume Imaging of Near-Native Cells and Tissues

(A) Cells cultured on EM grids are subjected to plunge freezing.

(B) Needle biopsy can be performed to extract tissues within 1–2 min, and immediately subjected to high pressure freezing.

(C) Serial cryoFIB/SEM is performed automatically for 5–20 h, depending on the volume to be imaged, dwell time, and average line count.

(D) A stack of 2D cryoSEM images enclosing the volume of the cell.

(E) 3D segmentation of the cell volume.

(F) Targeted cell lamella preparation on the intracellular region of interest identified by serial cryoFIB/SEM.

insulin-sensitive tissues), is a constituent of complex V required for its dimerization. Complex V ordinarily exists as a dimeric supercomplex required to shape the mitochondrial cristae, enabling efficient flow of the protons needed to fuel ATP synthesis. Recent cryoET of thin peripheral regions of LS patient cells harboring this *USMG5* gene mutation revealed significant disturbances in mitochondrial crista (Siegmund et al., 2018). The effect of the *USMG5* mutation on the level of whole-cell and subcellular architecture, however, has not been investigated.

Here, we developed and optimized a workflow using serial cryoFIB/SEM to study whole plunge-frozen primary fibroblast cells from a healthy individual and from an LS patient carrying the homozygous mutation in the *USMG5* gene previously shown to impair mitochondria cristae structure and ATP synthesis (Siegmund et al., 2018). The resulting 3D volumes of patient and control cells demonstrate a profound disruption of cellular and subcellular structures in LS patient cells. Compared with conventional serial FIB/SEM of stained and resin-embedded samples, serial cryoFIB/SEM offers a much faster (without a

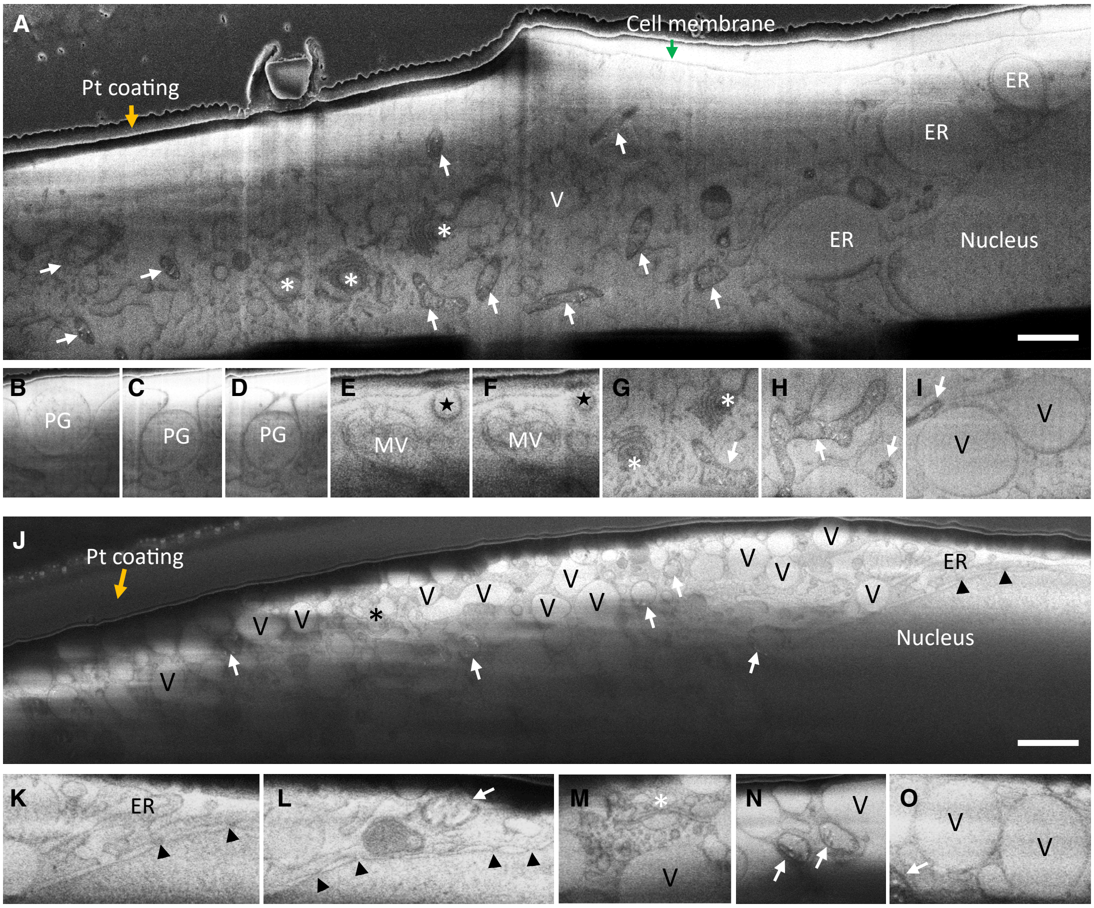

Figure 2. Serial cryoFIB/SEM of Frozen-Hydrated Primary Cells from Control and Patient Fibroblasts

(A) A representative cryoSEM image from a stack of 575 serial micrographs recorded from a control fibroblast cell cultured on an EM grid.

(B–I) An image gallery of subcellular structures and organelles observed in the control cell, including three consecutive slices of a phagosome entering the cell (B–D), two consecutive slices of an endosome (E and F, star), a multivesicular body (E and F, MV), Golgi complexes (G, asterisks), tubular-shaped mitochondria (G and H, arrow), and vacuoles (I and V).

(J) A representative cryoSEM image from a stack of 2018 serial micrographs recorded from a patient fibroblast cell cultured on an EM grid.

(K–O) An image gallery of subcellular structures and organelles observed in the patient cell, showing endoplasmic reticulum (K, ER), nuclear pores (K and L, arrowheads), Golgi complex (M, asterisk), mitochondria (L and N, arrows), and vacuoles (M, O, and V). Arrows, mitochondria; asterisks, Golgi; stars, endosome; PG, phagosome; MV, multivesicular body; V, vacuoles; arrowheads, nuclear pore; orange arrows, platinum GIS coating; green arrow, cell membrane; ER, endoplasmic reticulum. Scale bars, 1 μm.

lengthy dehydration and embedding process during sample preparation) and close-to-native technique for phenotypic characterization of whole cells or tissue, which could be exceedingly useful in clinical settings.

RESULTS

A Workflow for 3D Volume Imaging of Near-Native Cells and Tissues

To investigate the phenotypic impact of a specific *USMG5* gene mutation (c.87 + 1G > C) on cellular and subcellular structures in a near-native state, we cultured the primary fibroblast cells isolated from an LS patient and from a healthy individual on gold EM grids, which were subsequently plunge-frozen (Figure 1). Serial cryoFIB sectioning and cryoSEM imaging of frozen-hydrated primary fibroblast cells were performed using a Zeiss Crossbeam 550 instrument (Table S1). To maximize the cryoSEM image contrast and balance between resolution and total volume and time, we tested a number of FIB and SEM parameters, including pixel spacing, FIB slice thickness, FIB and SEM probe currents, acceleration potential, SEM dwell time, and average line count. Using a lateral pixel spacing of 10.5 nm for SEM imaging and a FIB slice thickness of 21.0 nm, an entire patient fibroblast cell was sliced through and 2018 slices were imaged at 4,096 × 3,072 pixels in about ~17.5 h, resulting in a total volume of 58,789 μm^3 (Video S1). For the control fibroblast cell, a similar voxel size was used, but a reduced raster of 3,072 × 1,150 pixels was used for imaging in total 575 slices. A total volume of 4,062 μm^3 was obtained within ~5.5 h for the control cell (Video S2). Detailed parameters are listed in Table S1. The density profile plots indicate that the resolution of cryoSEM images is at least 37 nm with 10 nm pixel size under the image conditions

CellPress
OPEN ACCESS

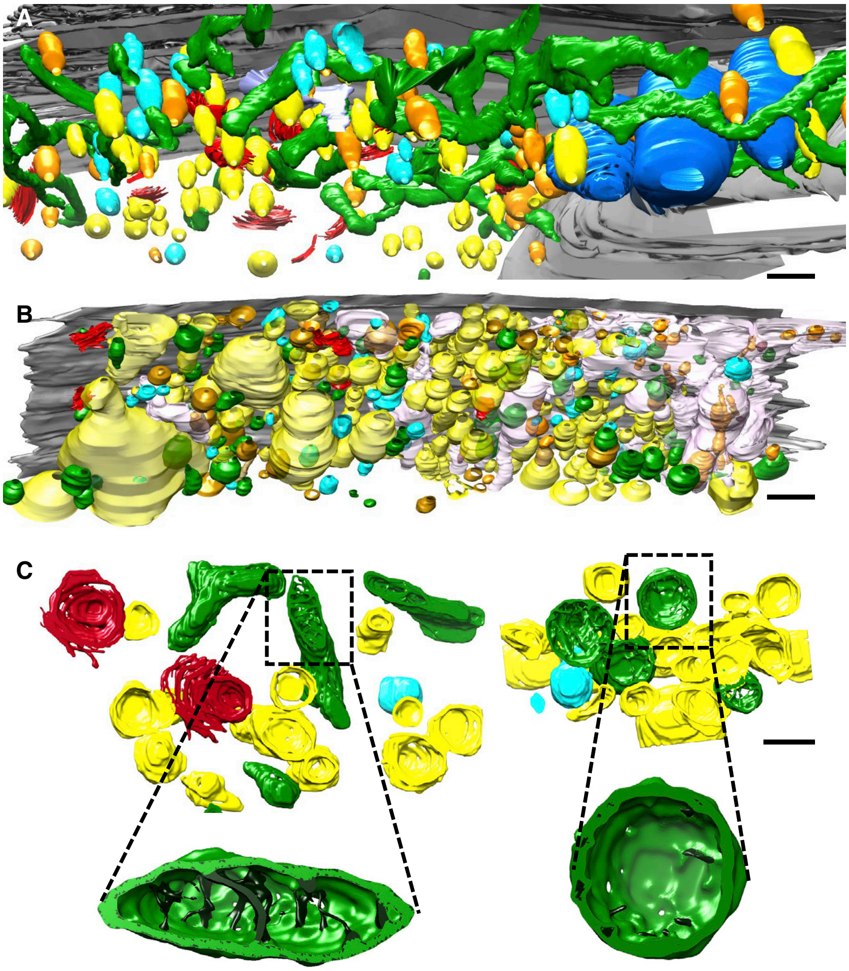

Figure 3. 3D Reconstruction and Segmentation of Control and Patient Cells

(A and B) Surface rendering of segmented volumes of control (A) and patient (B) fibroblast cells. Green, mitochondria; red, Golgi, yellow, vacuoles; orange, dense vesicles; cyan, partially dense vesicles; blue, ER.

(C) Segmentation of a small representative volume from control (left) and patient (right) fibroblasts. Inserts are enlarged views of a single mitochondrion in control (left) and patient (right) cells. Cristae are shown in dark green. Scale bars, 1 μm (A and B) and 500 nm (C).

specified (Figure S1). The actual resolution might be assessed using a crystalline material.

Serial cryoFIB/SEM Volume Imaging of Control and Patient Fibroblasts

Figure 2 shows cryoSEM images of representative slices from both patient and control cells. We noted a contrast imbalance between the lower part and the upper part of the cross-sectioned patient cell (Figure 2J). Since the cryoSEM image contrast is related to local surface potentials, the median potential (or threshold) can be different depending on the environment and local charge dissipation, such as the top cell region being close to the cold deposited platinum precursor layer and bottom part being in the vicinity of the support film and grid bars. In both cells, subcellular structures are clearly visible, especially membrane-enclosed subcellular compartments (Figures 2A and 2J), including the nucleus. Notably, serial cryoFIB/SEM provides unambiguous visualization of nuclear pores in human cells (Figures 2J–2L, arrowheads), which had not been achieved previously using this method. In the control cell, individual cellular organelles can be identified based on their distinct morphologies, including the cell membrane, a phagosome (Figures 2B–2D, PG, in three consecutive slices), endoplasmic reticulum, multivesicular bodies (Figures 2E and 2F, MV), Golgi (Figure 2G, *), mitochondria (Figures 2G–2I, white arrows), vacuole-like membranous structures (Figures 2I and 2V), and the cell nucleus. The LS patient fibroblast cell, however, shows substantial cytoarchitectural derangements, with the interior of the cell largely occupied by vacuolated structures of indeterminate origin. Of the residual identifiable structures, including mitochondria and Golgi, organelles are significantly decreased in volume and displayed gross morphological abnormalities. For example, the Golgi apparatus lacks extended membrane stacks (Figures 2J and 2M). More remarkably, compared with the complex shape and network of mitochondria in the control cell (Figures 2A, 2G, and 2H; Video S2), nearly all patient mitochondria are roughly round with minimal cristae (Figures 2J, 2L, and 2N; Video S1), consistent with our previous cryoET analyses (Siegmund et al., 2018). This suggests that the architecture responsible for energy metabolism in the patient cells is compromised, consistent with the earlier biochemical characterization of these primary fibroblasts (Barca et al., 2018). The patient cells also grow substantially slower than the control cells.

3D Reconstruction and Segmentation of Control and Patient Cells

For further analysis, we have performed 3D reconstruction and segmentation of the serial cryoFIB/SEM volume data for both patient and control cells (Figures 3A and 3B; Videos S1 and S2). In the volume rendering of the control cell, an extended network of oblate tubular-shaped mitochondria is evident (Figure 3A, green; Video S3), whereas the patient cell shows mitochondria that are discrete and mostly round or oval-shaped individuals (Figure 3B, green; Video S4). Some individual mitochondria appear in close association with one another in both control and patient cells (Figure S2). The size of vacuoles (Figure 3B, yellow) within the patient cell are also remarkably larger than those of the control cell, more abundant and densely packed (Figures 2A, 2J, 3A, and 3B). The overall volume of mitochondria and number of each organelle in patient and control cells are compared in Figure S3. To analyze the structural details of organelles, a small region of the cell was cropped and

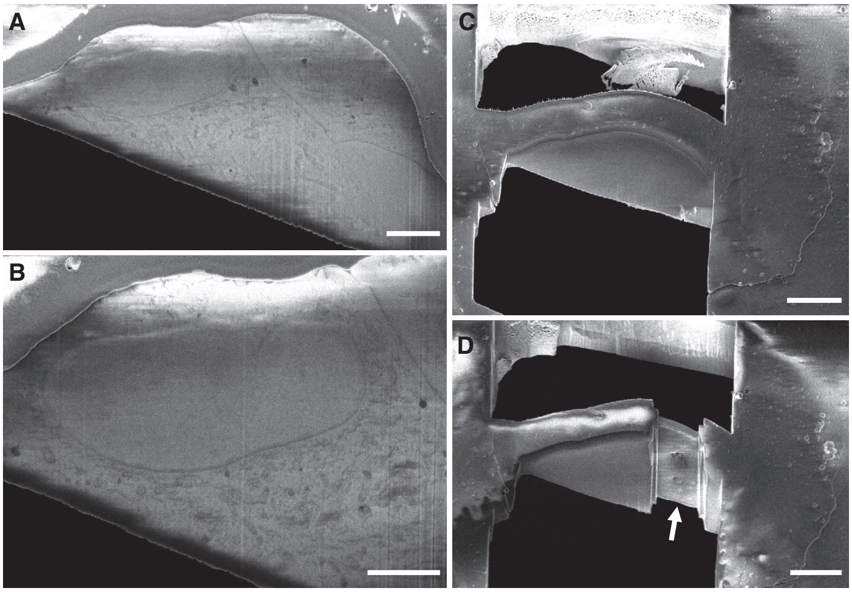

Figure 4. Targeted Cell Lamella Preparation by Serial cryoFIB/SEM

(A and B) Two representative cryoSEM images of cryoFIB block face, 320 nm apart.

(C and D) CryoSEM images of cell lamella preparation at the same region shown in (B). Arrow points to the thin lamella. Scale bars, 5 μm (A and B) and 10 μm (C and D).

segmented semi-automatically, as shown in Figures 3C and 3D (Videos S5 and S6). We can appreciate the drastic differences in the morphology of mitochondria and the shape and distribution of cristae between patient and control cells. Cristae structure is severely disturbed in the patient cell, appearing sparse in number and short, as previously observed by cryoET of limited regions of the cell periphery (Siegmund et al., 2018). The dramatic impact of complex V's failure to dimerize due to a specific *USMG5* gene mutation on overall cellular architecture and organelle structures in LS patient cells is now more fully appreciated in the greater context of the whole cell through *in situ* large volume imaging.

DISCUSSION

The majority of imaging studies on LS disease have been understandably focused on mitochondria. Nevertheless, our work demonstrates dramatic phenotypic changes to LS patient cells that extend beyond mitochondria to alter most, if not all, organelles within the cell, in particular a substantial accumulation of vacuoles. This is consistent with a recent study with PARL-deficient (*Parl*$^{-/-}$) mice exhibiting a Leigh-like syndrome, where, in addition to mitochondrial structural changes, similar intracellular vacuolization was observed in a response to alterations in complex III (Spinazzi et al., 2019). Using emerging serial cryoFIB/SEM technology, we captured and visualized an entire frozen-hydrated mammalian cell in 3D. More importantly, we applied this capability to studies of human disease cellular processes. This revealed a profound disruption of cellular and subcellular structures in a primary LS patient fibroblast cells. Such whole-cell volume phenotypic characterization of cells and tissues *in situ*, at the near-native state, offers an opportunity to improve our understanding of diseases beyond LS and potentially provides new means for clinical use, from diagnosis to treatment. The potential of combining large-scale cryo-volume imaging using serial cryoFIB/SEM, followed by cryoFIB lamella preparation of the specific region of interest identified through serial cryoFIB/SEM (Figure 4), with cryoET imaging of the targeted lamella at a high resolution on the exact same object, is especially exciting.

STAR★METHODS

Detailed methods are provided in the online version of this paper and include the following:

- KEY RESOURCES TABLE
- RESOURCE AVAILABILITY
  - Lead Contact
  - Materials Availability
  - Data and Code Availability
- EXPERIMENTAL MODEL AND SUBJECT DETAILS
  - Ethics Statement
  - Cell Lines
  - Primary Cell Cultures
- METHODS DETAILS
  - Sample Preparation
  - Serial cryoFIB/SEM
  - Local Reconstruction and Subvolume Segmentation
  - Global Image Alignment and 3D Modelling
- QUANTIFICATION AND STATISTICAL ANALYSIS

SUPPLEMENTAL INFORMATION

Supplemental Information can be found online at https://doi.org/10.1016/j.str.2020.10.003.

ACKNOWLEDGMENTS

We thank Drs. Teresa Brosenitsch and Luiza Mendonça for critical reading of the manuscript. We are grateful to Dr. Michio Hirano for providing the control and patient cells. We also thank Drs. James Gilchrist for technical support, and Drs. Tao Ni, Min Xu, Julika Radecke, and Andrew Howe for helpful discussions about segmentation. We thank Dr. Saskia Mimietz-Oeckler and Andreas Halladay, Leica Microsystems, for technical support. This work was supported by

CellPress
OPEN ACCESS

the Department of Defense grants PR141292 (to Z.F.) and PR192466 (to Z.F.), the NIH P50 grant AI150481 (to P.Z.), the UK Wellcome Trust Investigator Award 206422/Z/17/Z (to P.Z.), and the UK Biotechnology and Biological Sciences Research Council grant BB/S003339/1 (to P.Z.).

AUTHOR CONTRIBUTIONS

P.Z. conceived and designed the experiments. J.N. cultured cells and X.F. prepared cryo-specimens. A.S., L.C.Z.-D., and M.L.M.-F. performed serial cryo-FIB/SEM. Y.Z., P.P.G., A.M.W., and D.S. performed segmentation and, with P.Z., analyzed data. Y.Z. and P.Z. wrote the paper with support from other authors.

DECLARATION OF INTERESTS

The authors declare no competing interests.

Received: June 26, 2020
Revised: August 31, 2020
Accepted: October 5, 2020
Published: October 22, 2020

REFERENCES

Akiva, A., Nelkenbaum, O., Schertel, A., Yaniv, K., Weiner, S., and Addadi, L. (2019). Intercellular pathways from the vasculature to the forming bone in the zebrafish larval caudal fin: possible role in bone formation. J. Struct. Biol. *206*, 139–148.

Barca, E., Ganetzky, R.D., Potluri, P., Juanola-Falgarona, M., Gai, X., Li, D., Jalas, C., Hirsch, Y., Emmanuele, V., Tadesse, S., et al. (2018). USMG5 Ashkenazi Jewish founder mutation impairs mitochondrial complex V dimerization and ATP synthesis. Hum. Mol. Genet. *27*, 3305–3312.

Cardona, A., Saalfeld, S., Schindelin, J., Arganda-Carreras, I., Preibisch, S., Longair, M., Tomancak, P., Hartenstein, V., and Douglas, R.J. (2012). TrakEM2 software for neural circuit reconstruction. PLoS One *7*, e38011.

Chang, X., Wu, Y., Zhou, J., Meng, H., Zhang, W., and Guo, J. (2020). A meta-analysis and systematic review of Leigh syndrome: clinical manifestations, respiratory chain enzyme complex deficiency, and gene mutations. Medicine (Baltimore) *99*, e18634.

Himes, B.A., and Zhang, P. (2018). emClarity: software for high-resolution cryo-electron tomography and subtomogram averaging. Nat. Methods *15*, 955–961.

Kremer, J.R., Mastronarde, D.N., and MacIntosh, J.R. (1996). Computer visualization of three-dimensional image data using IMOD. J. Struct. Biol. *116*, 71–76.

Kizilyaprak, C., Stierhof, Y.D., and Humbel, B.M. (2019). Volume microscopy in biology: FIB-SEM tomography. Tissue Cell *57*, 123–128.

Lake, N.J., Bird, M.J., Isohanni, P., and Paetau, A. (2015). Leigh syndrome: neuropathology and pathogenesis. J. Neuropathol. Exp. Neurol. *74*, 482–492.

Lee, J.S., Kim, H., Lim, B.C., Hwang, H., Choi, J., Kim, K.J., Hwang, Y.S., and Chae, J.H. (2016). Leigh syndrome in childhood: neurologic progression and functional outcome. J. Clin. Neurol. *12*, 181–187.

Lowe, D.G. (2004). Distinctive image features from scale-invariant keypoints. Int. J. Comput. Vis. *60*, 91–110.

Lucic, V., Rigort, A., and Baumeister, W. (2013). Cryo-electron tomography: the challenge of doing structural biology in situ. J. Cell Biol. *202*, 407–419.

Makino, M., Horai, S., Goto, Y., and Nonaka, I. (2000). Mitochondrial DNA mutations in Leigh syndrome and their phylogenetic implications. J. Hum. Genet. *45*, 69–75.

McCormick, E.M., Zolkipli-Cunningham, Z., and Falk, M.J. (2018). Mitochondrial disease genetics update: recent insights into the molecular diagnosis and expanding phenotype of primary mitochondrial disease. Curr. Opin. Pediatr. *30*, 714–724.

Pettersen, E.F., Goddard, T.D., Huang, C.C., Couch, G.S., Greenblatt, D.M., Meng, E.C., and Ferrin, T.E. (2004). UCSF Chimera—a visualization system for exploratory research and analysis. J. Comput. Chem. *25*, 1605–1612.

Pronicki, M., Matyja, E., Piekutowska-Abramczuk, D., Szymanska-Debinska, T., Karkucinska-Wieckowska, A., Karczmarewicz, E., Grajkowska, W., Kmiec, T., Popowska, E., and Sykut-Cegielska, J. (2008). Light and electron microscopy characteristics of the muscle of patients with SURF1 gene mutations associated with Leigh disease. J. Clin. Pathol. *61*, 460–466.

Schertel, A., Snaidero, N., Han, H.M., Ruhwedel, T., Laue, M., Grabenbauer, M., and Mobius, W. (2013). Cryo FIB-SEM: volume imaging of cellular ultrastructure in native frozen specimens. J. Struct. Biol. *184*, 355–360.

Schirra, R.T., Jr., and Zhang, P. (2014). Correlative fluorescence and electron microscopy. Curr. Protoc. Cytom. *70*, 12 36 11–10.

Siegmund, S.E., Grassucci, R., Carter, S.D., Barca, E., Farino, Z.J., Juanola-Falgarona, M., Zhang, P.J., Tanji, K., Hirano, M., Schon, E.A., et al. (2018). Three-dimensional analysis of mitochondrial crista ultrastructure in a patient with Leigh syndrome by in situ cryoelectron tomography. Iscience *6*, 83.

Sofou, K., De Coo, I.F., Isohanni, P., Ostergaard, E., Naess, K., De Meirleir, L., Tzoulis, C., Uusimaa, J., De Angst, I.B., Lonnqvist, T., et al. (2014). A multicenter study on Leigh syndrome: disease course and predictors of survival. Orphanet J. Rare Dis. *9*, 52.

Spinazzi, M., Radaelli, E., Horre, K., Arranz, A.M., Gounko, N.V., Agostinis, P., Maia, T.M., Impens, F., Morais, V.A., Lopez-Lluch, G., et al. (2019). PARL deficiency in mouse causes complex III defects, coenzyme Q depletion, and Leigh-like syndrome. Proc. Natl. Acad. Sci. U S A *116*, 277–286.

Steyer, A.M., Schertel, A., Nardis, C., and Mobius, W. (2019). FIB-SEM of mouse nervous tissue: fast and slow sample preparation. Methods Cell Biol. *152*, 1–21.

Sutton, G., Sun, D., Fu, X., Kotecha, A., Hecksel, C.W., Clare, D.K., Zhang, P., Stuart, D.I., and Boyce, M. (2020). Assembly intermediates of orthoreovirus captured in the cell. BioRxiv. https://doi.org/10.1101/2020.06.10.144998.

Sviben, S., Gal, A., Hood, M.A., Bertinetti, L., Politi, Y., Bennet, M., Krishnamoorthy, P., Schertel, A., Wirth, R., Sorrentino, A., et al. (2016). A vacuole-like compartment concentrates a disordered calcium phase in a key coccolithophorid alga. Nat. Commun. *7*, 11228.

Vidavsky, N., Addadi, S., Schertel, A., Ben-Ezra, D., Shpigel, M., Addadi, L., and Weiner, S. (2016). Calcium transport into the cells of the sea urchin larva in relation to spicule formation. Proc. Natl. Acad. Sci. U S A *113*, 12637–12642.

Vidavsky, N., Masic, A., Schertel, A., Weiner, S., and Addadi, L. (2015). Mineral-bearing vesicle transport in sea urchin embryos. J. Struct. Biol. *192*, 358–365.

Wang, K., Strunk, K., Zhao, G., Gray, J.L., and Zhang, P. (2012). 3D structure determination of native mammalian cells using cryo-FIB and cryo-electron tomography. J. Struct. Biol. *180*, 318–326.

Wu, G.H., Mitchell, P.G., Galaz-Montoya, J.G., Hecksel, C.W., Sontag, E.M., Gangadharan, V., Marshman, J., Mankus, D., Bisher, M.E., Lytton-Jean, A.K.R., et al. (2020). Multi-scale 3D cryo-correlative microscopy for vitrified cells. Structure. https://doi.org/10.1016/j.str.2020.07.017.

Zhang, P. (2019). Advances in cryo-electron tomography and subtomogram averaging and classification. Curr. Opin. Struct. Biol. *58*, 249–258.

STAR★METHODS

KEY RESOURCES TABLE

REAGENT or RESOURCE	SOURCE	IDENTIFIER
Biological Samples		
Fetal bovine serum	Sigma-Aldrich	12103C
Chemicals, Peptides, and Recombinant Proteins		
MEM Vitamin solution	Thermo Fisher	11120037
Antibiotic-antimycotic	Thermo Fisher	15240062
Dulbecco's minimal essential media (DMEM)	Thermo Fisher	11965092
Fibronectin	Sigma-Aldrich	33016015
Deposited Data		
Serial cryoFIB/SEM raw images	This Paper	EMPIAR-10515
Experimental Models: Cell Lines		
Primary skin fibroblasts from a patient carrying a homozygous USMG5 mutation (C.87+1G>C, 1 base pair after the end of Exon 3)	Siegmund et al., 2018	N/A
Primary control cells from healthy human subjects	Siegmund et al., 2018	N/A
Software and Algorithms		
IMOD	Kremer et al., 1996	https://bio3d.colorado.edu/imod/
MATLAB	MathWorks	https://www.mathworks.com/products/matlab.html
Chimera	Pettersen et al., 2004	https://www.cgl.ucsf.edu/chimera/
TrakEM2	Cardona et al., 2012	https://imagej.net/TrakEM2
Other		
Quantifoil Gold R2/2 grids, 300 mesh	Quantifoil	Q350AR2
Glass-bottom culture dishes	MatTek Corporation	P35G-1.5-7-C

RESOURCE AVAILABILITY

Lead Contact
Further information and requests should be directed and will be fulfilled by the Lead Contact, Prof. Peijun Zhang, (peijun@strubi.ox.ac.uk).

Materials Availability
This study did not generate new unique reagents.

Data and Code Availability
The raw Serial cryoFIB/SEM images during this study has been deposited at EMPIAR (https://www.ebi.ac.uk/pdbe/emdb/empiar/) with the access code EMPIAR-10515.

EXPERIMENTAL MODEL AND SUBJECT DETAILS

Ethics Statement
The research received approval from the MRC Regulatory Support Centre.

Cell Lines
Skin fibroblasts from a patient carrying a homozygous USMG5 mutation (C.87+1G>C, 1 base pair after the end of Exon 3), and control cells from both male and female healthy human subjects are provided by Dr. Michio Hirano, Columbia University. Cell lines have not been authenticated.

Primary Cell Cultures

Cells were cultured in Dulbecco's minimal essential media (DMEM) (Thermo Fisher, Waltham, MA, USA) supplemented with 15% fetal bovine serum (FBS) (Sigma-Aldrich, St. Louis, MO, USA), 1% vitamin solution and 1% antibiotic-antimycotic (Thermo Fisher) as described earlier(Siegmund et al., 2018).

METHODS DETAILS

Sample Preparation

All experiments were conducted on cells cultured for <15 passages. Cells were plated onto gold R2/2 Quantifoil finder EM grids (Quantifoil Micro Tools GmbH, Jena, Germany) at density of 0.5-1 × 10^5 cells/ml (total 2 ml culture) in glass-bottom culture dishes (MatTek Corporation, Ashland, MA). The gold EM grids were coated with 50 μg/ml fibronectin (Sigma-Aldrich) and sterilized under UV light for 2 hours before use. For the control cells, after 48 hours culture, the grids were blotted with a filter paper and plunge-frozen into liquid ethane for rapid vitrification using an FEI Vitrobot (FEI, Hillsboro, OR) at ~100% humidity. Patient cells grew slowly and were cultured for 5 days before plunge-freezing.

Serial cryoFIB/SEM

For patient cells, the plunge-frozen EM grid was mounted in a Leica Vacuum Cryo Manipulation (VCM) preparation box (Leica Microsystems GmbH, Vienna, Austria) on a Leica cryo-holder for freeze-fracturing under cryogenic conditions. The TEM grid was held down on the flat top surface by using the clamp. The sample holder was transferred into a Leica ACE 600 cryo-sputter coater using a Leica VCT500 sample shuttle (Leica Microsystems, Vienna, Austria). At -154°C, the sample was sputter-coated with a 4 nm thick tungsten layer. The samples were then transferred into a ZEISS Crossbeam 550 FIB-SEM (Carl Zeiss Microscopy GmbH, Oberkochen, Germany). The cryo-stage temperature was maintained at -155°C and the system vacuum was 1.6×10^{-6} mbar. For areas containing cells, a cold deposition of platinum precursor material was achieved by opening the gas valve for 45 seconds. For cold deposition, the gas reservoir temperature was 28°C, and the distance between the gas capillary and the sample was about 3 mm.

First, a viewing channel for SEM imaging was milled using a FIB-milling probe current of 7 nA. The resulting cross-section was polished with a FIB probe current of 3 nA. For serial sectioning and imaging, a FIB probe current of 700 pA was used, and the FIB slice thickness was 21 nm. SEM images using InLens SE detection with a SEM probe current of 35 pA, a SEM acceleration potential of 2.3 keV and a dwell time of 100 ns were recorded. The lateral pixel spacing for SEM imaging was 10.5 nm and the image size was 4096 × 3072 pixels. Line Average with a line average count N = 61 was used for noise reduction.

The control cell sample (bare-TEM grid clipped into an autogrid ring) was mounted on a pre-tilted Leica sample holder for on-grid-thinning (Leica Microsystems GmbH, Vienna, Austria). After sputter-coating and transfer into the Crossbeam 550 FIB/SEM, a cold deposition of platinum precursor was done following the same procedure as above. The Crossbeam 550 system pressure was 8×10^{-7} mbar. A FIB probe current of 700 pA and a FIB slice thickness of 20 nm were used for serial sectioning and imaging. The lateral pixel spacing for SEM imaging was 10 nm and the imaging box was reduced to 3072 × 1150 pixel since the cell width is much larger than its height. For SEM imaging, InLens SE detection, a SEM probe current of 59 pA, a SEM acceleration potential of 1.9 keV and a dwell time of 200 ns were used. We employed a line average count N = 19 was used for noise reduction.

For cryoSEM imaging, the acceleration potential influences sample charging and is chosen in order to minimize sample charging and charging artefacts at interfaces. Due to the different mounting geometry, sample charging conditions are affected, and the acceleration potential is varied to optimize for imaging conditions. Since a charging and beam sensitive sample is imaged, short dwell times are advantageous and the total electron dose (beam current * dwell time * line average count) is restricted to minimize beam damage. By variation of how a specific electron dose is provided the imaging conditions are optimized especially near interfaces.

Local Reconstruction and Subvolume Segmentation

The raw SEM images were first aligned and a 3D volume generated using IMOD(Kremer et al., 1996). Subvolumes with representative features were cropped out from each aligned image stack (300×200 pixel × 40 slices or ~3.2 × 2.1 × 0.84 μm for patient cell and 500×400 pixel×40 slices or ~5.0 × 4.0 × 0.8 μm for control cell). The alignment between images was refined using an in-house Matlab script based on the Imregister function. To make objects smooth, 19 additional images were generated, with a linear interpolation, and inserted between two successive image slices with a home-made Matlab script. Using PixelAnnotationTool available online (https://github.com/abreheret/PixelAnnotationTool/releases), specific organelles, such as mitochondria, Golgi etc., were labeled manually from the cropped images as masks, which were then used to extract the image data from the corresponding region separately. These segmented image volumes were displayed in Chimera(Pettersen et al., 2004).

Global Image Alignment and 3D Modelling

The region containing the cell content was masked using Chimera software(Pettersen et al., 2004) with a boundary contour that was generated in 3dmod to just include the cell. The masked image slices (237 slices from the control cell and 218 slices from the patient cell) were first aligned with the Tiltxcorr(Kremer et al., 1996) program in IMOD using cross-correlation to determine the X and Y translations between successive image slices. The coarse-aligned image stacks were registered further using a SIFT-based

algorithm(Lowe, 2004) adapted to run on TrakEM2 plugin for FIJI(Cardona et al., 2012). The post-registration images were exported into 3dmod(Kremer et al., 1996) for further manual segmentation. Chimera was used for display of segmented 3D models.

QUANTIFICATION AND STATISTICAL ANALYSIS

The parameters of serial cryoFIB/SEM in Table S1 are optimal imaging conditions. Resolution assessment was done through density profile plot in Digital Micrograph (Gatan Inc.) software and presented in Figure S1. The numbers for specific organelles were counted from the segmented cells and presented in Figure S3. The volumes for segmented mitochondria were calculated in IMOD and presented in Figure S3.

Structure, Volume *29*

Supplemental Information

Serial cryoFIB/SEM Reveals Cytoarchitectural Disruptions in Leigh Syndrome Patient Cells

Yanan Zhu, Dapeng Sun, Andreas Schertel, Jiying Ning, Xiaofeng Fu, Pam Pam Gwo, Alan M. Watson, Laura C. Zanetti-Domingues, Marisa L. Martin-Fernandez, Zachary Freyberg, and Peijun Zhang

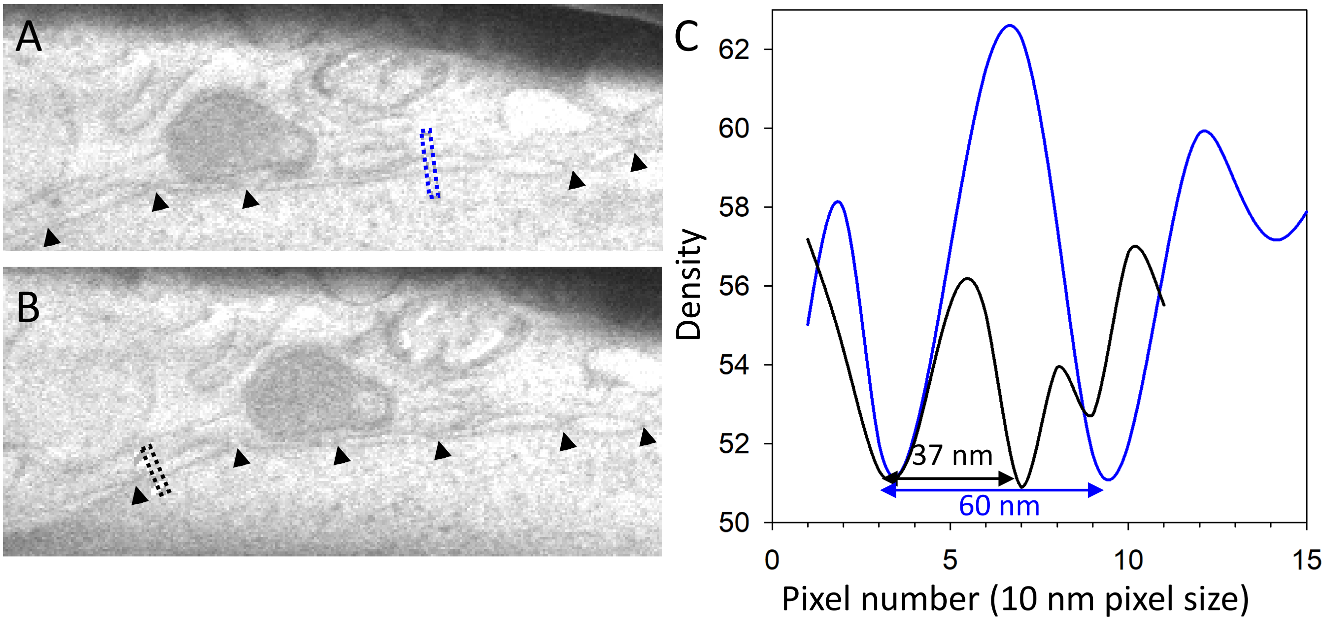

Figure S1 | Resolution assessment. A-B) Representative cryoSEM images from a stack of 2018 serial micrographs recorded from a patient fibroblast cell. Black arrow heads indicate nuclear pores. Blue and black boxes enclose areas for density profile. C) Density profile plots of the nuclear membranes. The blue line from the area shown in (A) and the black line from the area shown in (B). The separation between the two membranes is 37 nm in (B). Related to Figure 2.

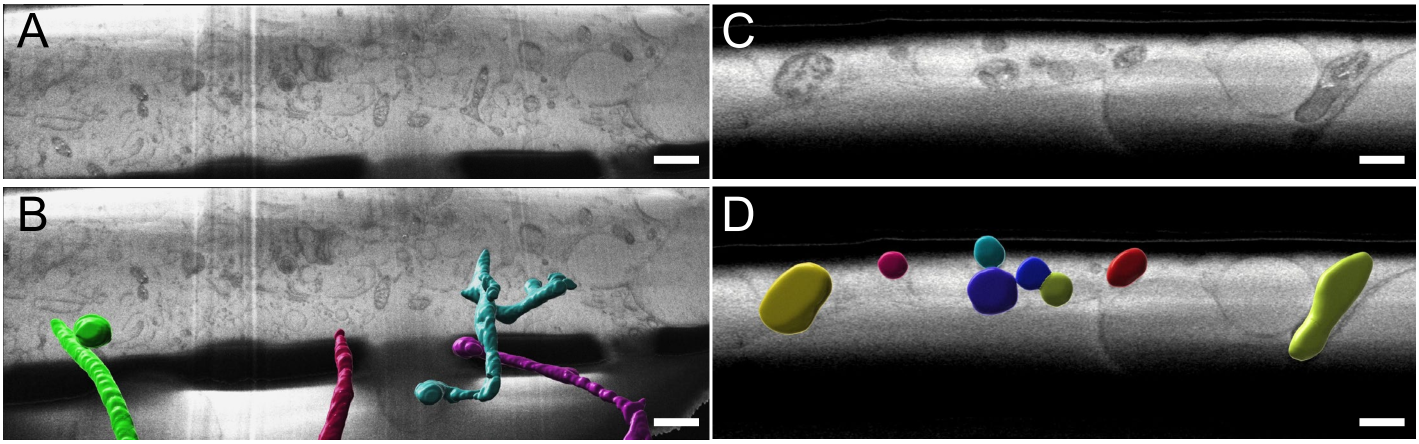

Figure S2 | Close association of mitochondria in control (A-B) and patient (C-D) cells. Mitochondria were manually segmented from control (A) and patient (C) datasets using NIS Elements (Nikon). The segmented images were overlaid with the RAW data in Imaris 9.5 (Bitplane) where the segmented areas were transformed into objects using surfacing tools (B and D, also see Movie S7 and S8). Independent objects are signified by a unique color. Scale bars, 1 µm in a & b, 0.3 µm in C & D. Related to Figure 3.

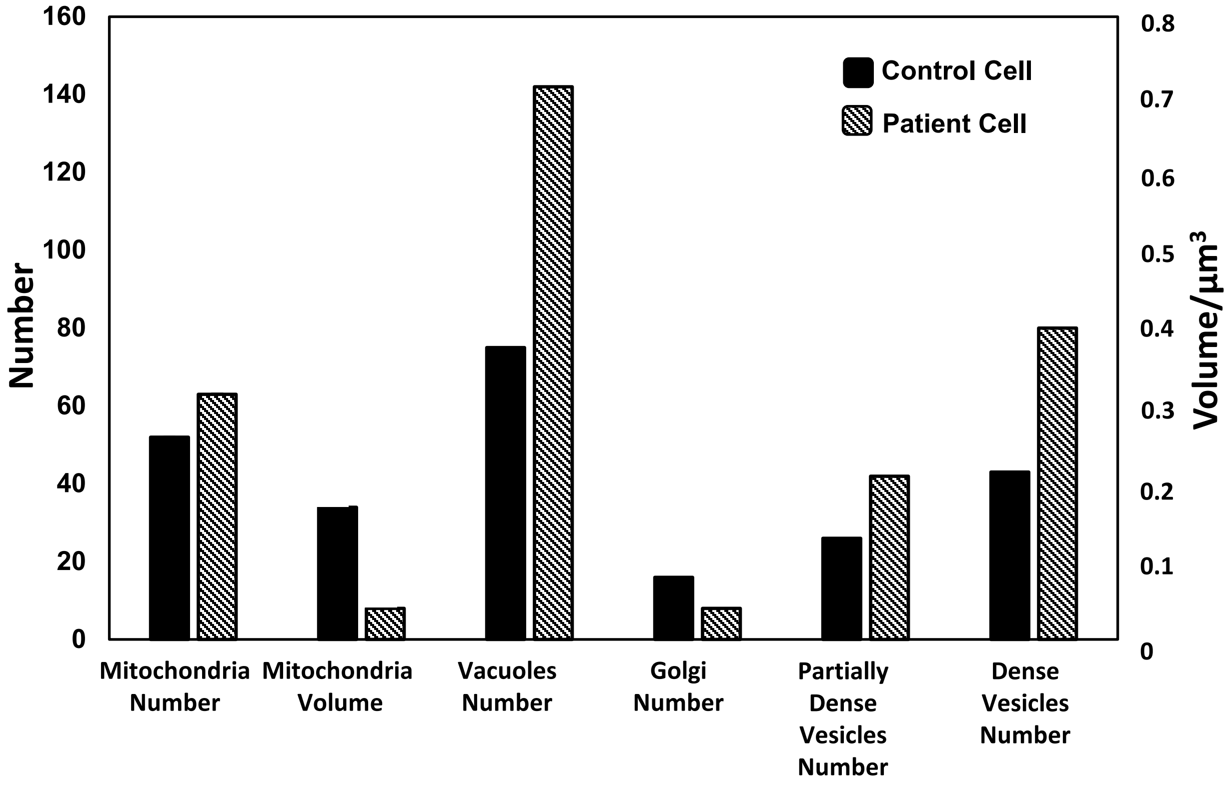

Figure S3 | Number and volume of different organelles in control and patient cells. Related to Figure 3.

Table S1: Summary of serial cryoFIB/SEM parameters. Related to STAR☆Methods, Methods Details, Serial cryoFIB/SEM section.

	FIB Probe current (pA)	SEM Probe current (pA)	SEM acceleration potential (keV)	Dose pA*nS /Å²	Dwell time (ns)	Line averagin g count	Pixel size x-y-z (nm)	Image size x-y (pixel)	No. Slices	Volume (µm³)	Time (h:m)
**Patient**	700	35	2.3	21.3	100	61	10.5 x 10.5 x 21.0	4096 × 3072	2018	50,784	17:20
**Control**	700	59	1.9	22.4	200	19	10.0 x 10.0 x 20.0	3072 × 1150	575	4,062	5:40
